# Origin of Magnetically Induced Optical Transmission of Magnetic Nanocomposite Films

**DOI:** 10.3390/polym12112533

**Published:** 2020-10-29

**Authors:** Qiushu Zhang, Bei Peng, Jintao Xu, Mengqi Chu

**Affiliations:** School of Mechanical and Electrical Engineering, University of Electronic Science and Technology of China, Chengdu 611731, China; qiushuzhang@uestc.edu.cn (Q.Z.); xujintao080215@163.com (J.X.); mengqi_cuestc@163.com (M.C.)

**Keywords:** optical transmission, nanocomposite, magnetic films, magnetostriction

## Abstract

Herein, we present an investigation on the origin of the magnetically induced optical transmission of composite films comprised of polydimethylsiloxane and magnetic nanofillers via experiment and simulation. Structured and unstructured films were used in the study, which were fabricated with and without magnetic fields, respectively. Altered optical transmittance was observed from both types of films when they were subjected to an external magnetic field. Numerical analyses were performed to investigate the effect of the particle movement under magnetic field and the film magnetostriction on the film optical transmittance. The simulation results show that the changed light transmission under magnetic field is mainly due to a variation in the film thickness resulting from the film magnetostriction. The ellipsometric analysis results confirm the altered film thickness in response to the external magnetic field, and the measurements of the film magnetostrictive stresses validate that there is magnetostriction in the magnetic composite films. Additionally, it is indicated that there might be some relationship between the magnetically induced optical transmission and the film magnetostrictive stress under certain conditions.

## 1. Introduction

Polydimethylsiloxane (PDMS), a silicone elastomer, has many interesting material properties, and therefore finds widely diverse scientific and technological applications. For example, it is used as a replication mold in micro/nanomachining technology owing to its very good contour accuracy (<10 nm) [1,2]. It is often found in biological experiments and in clinical practice due to its nontoxicity and biocompatibility [3,4]. It is also frequently used to fabricate microfluidic devices and other micro/nano devices [5,6]. Besides, it has high transparency in the visible spectrum and extremely low autofluorescence. Hence, it is a suitable material for optical components bearing some microstructures or nanostructures such as lenses, waveplates, waveguides, and gratings [7,8,9,10].

In addition, PDMS can serve as matrix material of magnetorheological elastomers (MREs) that are typically comprised of magnetic particles dispersed in a nonmagnetic elastic matrix. As a promising class of smart materials, MREs can respond to external stimuli such as a magnetic field, force, or temperature and thus have great potential for application in the engineering fields [11,12,13]. The studies of PDMS-based MREs were mainly concentrated on their mechanical properties [14,15,16,17,18]. Relatively less attention has been given to their optical properties. The investigation on this subject might open a way to extend the applications of MREs to the field of optics. In our previous work, MRE films consisting of PDMS and magnetic nanoparticles were prepared and optically characterized [19]. The experimental results indicate that the transparency of the MRE films can be altered via applying an external magnetic field, which is tentatively ascribed to the film deformation under magnetic field. However, continued efforts are still highly required to obtain an in-depth understanding of the origin of the magnetically induced light transmission of the magnetic composite films.

Magnetically induced optical transmission has been observed from magnetic fluids that are suspensions of magnetic nanosized particles dispersed in a liquid medium [20,21,22,23,24]. When a magnetic field is applied to magnetic fluid, magnetic nanoparticles in the magnetic fluid would form a chain-like structure. The microstructure of the magnetic fluid would change with the applied magnetic field, which leads to a variation in the optical transmittance of the magnetic fluid. The researchers have explained the magnetically induced light transmission of the magnetic fluids by the field-induced change in the particles’ overlap from the geometric shadowing effect or the field-induced variation in the area occupied by the relatively optic transparent liquid phase [21,23]. Nevertheless, it is quite different in the case of MREs. Under a magnetic field, it is much harder for magnetic particles to move in MREs than in magnetic fluids. However, applying an external magnetic field would bring about a deformation of MREs due to the complex magnetic interactions between particles and mechanical interactions between particles and elastic matrix [25,26,27].

The focus of this study is on the origin of the magnetic-field-induced optical transmittance of the magnetic nanocomposite films composed of PDMS and magnetic nanofillers. The samples used in the research include Fe_3_O_4_-PDMS and Fe-PDMS composite films with varying nanoparticle loadings that were prepared by spin casting onto the substrates. The films with structured and unstructured nanoparticle distributions were fabricated in the presence and absence of an external magnetic field, respectively. The experimental data indicate that the application of the magnetic field significantly changes the light transmission of the films. The effect of the particle movement under magnetic field and the film magnetostriction on the film optical transmission was investigated by 3D (three-dimensional) and 2D numerical modelings, respectively. The simulation results show that the magnetic field-induced variation in the film optical transmittance mainly arises from the film magnetostriction that results in a variation in the film thickness. The ellipsometric analysis results validate the changed film thickness caused by the magnetic field, and the measurement of the film stresses under magnetic field reveals that there are magnetostrictive stresses, and thus magnetostriction in the composite films. These experimental results further verify the simulation results. In addition, the experimental results imply that the magnetically induced optical transmission is probably related to the film magnetostrictive stress under certain conditions.

## 2. Materials and Methods

The composite films for the measurement of the optical transmittance were fabricated using Fe_3_O_4_ (20 nm in diameter) or Fe (50 nm in diameter) nanoparticles incorporated as fillers in PDMS elastomer. The nanoparticles are provided by Beijing DK Nano Technology Co., Ltd., Beijing, China. PDMS used for the samples is a two-part kit (Sylgard 184, Dow Corning, Midland, TX, USA), containing silicone elastomer base and cross linker agent. In the process of the composite film preparation, the PDMS matrix was produced by mixing the silicone elastomer base and the cross linker agent in proportions of 10:1 (*w*/*w*) at room temperature. The organic solvent toluene was then added to the PDMS mixture in proportions of 1:1 (*w*/*w*). Following that, the mixture solutions were loaded with magnetic nanofillers whose weight fractions were 1, 3, 5, 7, and 10 wt%, respectively. After each mixing, ultrasonically stirring was carried out for the purpose of removal of trapped air and sufficient mixing of components. The resulting solutions were then spin coated onto the quartz slices to form the composite films. For the unstructured samples, the films were directly thermally cured to complete the crosslinking process. For the structured samples, the films were placed in a uniform magnetic field (B = ~300 mT) for 2 min to produce structured nanoparticle distributions before they were cured. The magnetic field was applied with its direction parallel to the film surface. All the films were of about 10 µm thickness. More details on the composite film fabrication can be seen in the previous paper [19].

In the experimental setup for measuring the optical transmittance of the composite films, the incident light source is a semiconductor laser with a wavelength of 650 nm and a power of 3 mW. The intensity of transmitted light is converted into an electric signal by a photodiode and then the signal is amplified. The optical transmittance of every sample was measured without applying magnetic field, as well as under a homogenous magnetic field (B = ~300 mT). The applied magnetic field was created by two parallel permanent magnets, between which the sample was placed in such a way that the direction of the magnetic field was parallel to the film surface. To ensure the homogeneity of the applied magnetic field, the samples were placed in the central zone of the magnetic field. The influence of magnetic field on the thickness of the composite films was investigated through an ellipsometer (SE 850, SENTECH Instruments GmbH, Berlin, Germany). The film thickness of the sample was measured both without and with application of a magnetic field. When measuring the film thickness under magnetic field, a uniform magnetic field (B = ~300 mT) was created and applied to the films in the same way as the film transmittance testing.

The film magnetostrictive stresses were investigated by thin film stress measurement system (Toho technology, FLX-2320-S, Nagoya, Japan). A structure was designed and made to hold sample and apply magnetic field. The samples were fabricated by using the preparation process similar to that described above except that Si wafer was utilized as substrate instead of quartz slice. Si was selected as substrate material as it is well characterized and is fabricated with high precision. The film stress under magnetic field was calculated by Stoney’s equation,

(1) $$\sigma =\left(\frac{E_{s}}{1-v_{s}}\right)\frac{t_{s}^{2}}{6t_{f}}\left(\frac{1}{R_{a}}-\frac{1}{R_{b}}\right),$$

where *E_s_*, vs. and *t_s_* are the Young’s modulus, Poisson’s ratio and thickness of the substrate Si, *t_f_* the thickness of the composite films, and *R_a_* and *R_b_* the curvature of the wafers after and before the application of the magnetic field [28,29]. The radius of curvature was measured by the FLX-2320-S machine based on the change in angle (incident vs. reflected) of a rastered laser. During the measurement process, diametric scans were performed at 50 intervals. A homogenous magnetic field of B = ~300 mT was generated and imposed on the films in the same way as the measurement of the film optical transmittance.

## 3. Results and Discussion

**Figure 1:**
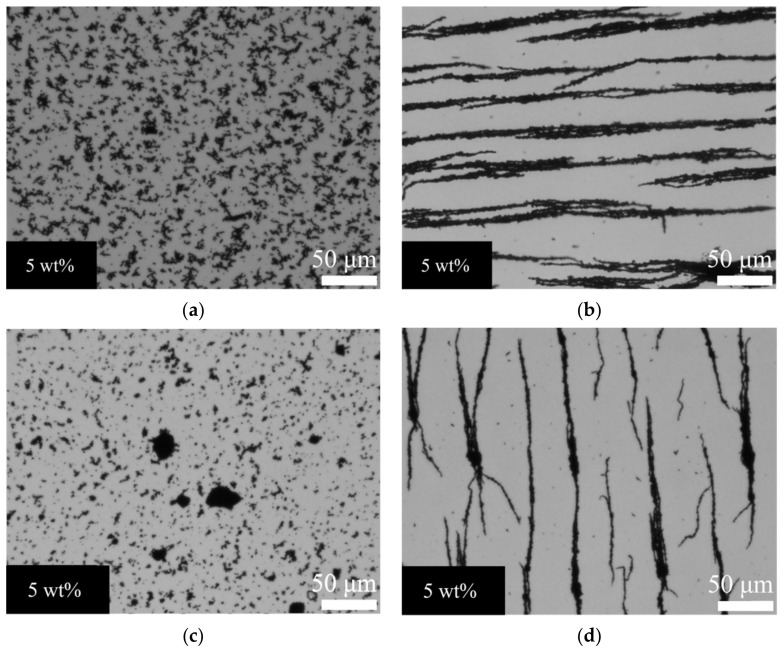
Optical micrographs of the composite films consisting of PDMS and 5 wt% magnetic nanoparticles: (**a**) unstructured Fe_3_O_4_-PDMS sample, (**b**) structured Fe_3_O_4_-PDMS sample, (**c**) unstructured Fe-PDMS sample, and (**d**) structured Fe-PDMS sample. A homogenous magnetic field of B = 300 mT was imposed during the fabrication of the structured samples. Magnification: 200×.

Figure 1 shows the microstructure pictures of the Fe_3_O_4_–PDMS and Fe–PDMS composite films with 5 wt% of magnetic nanofillers. In the unstructured samples, the particle fractal structures are randomly dispersed in the PDMS matrix. On the other hand, the particle columnlike structures are seen in the structured films, which were formed by magnetically aligning during the film preparation. Other unstructured and structured films have microstructures similar to those displayed in Figure 1.

**Figure 2:**
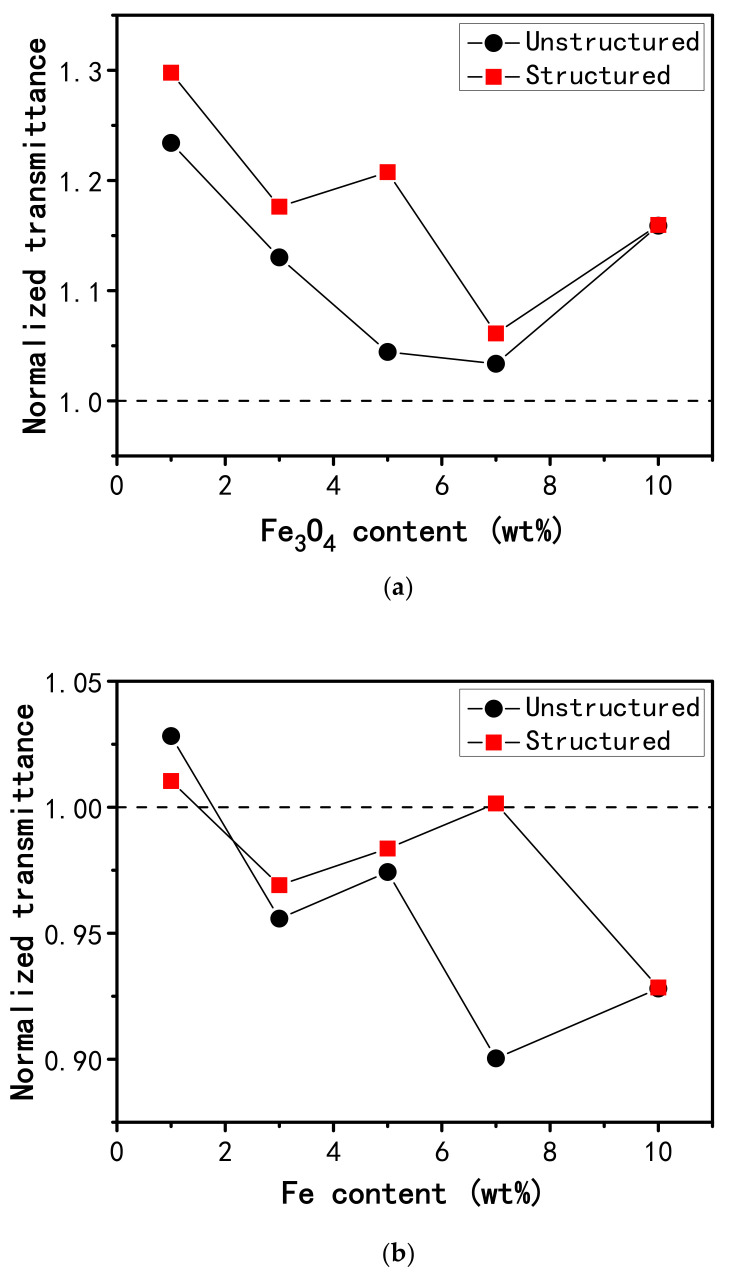
Film transmittance under magnetic field plotted as a function of nanoparticle loading for the unstructured (circles) and structured (squares) samples: (**a**) Fe_3_O_4_-PDMS films, and (**b**) Fe-PDMS films.

Figure 2 shows the particle loading dependence of the normalized transmittance with an applied magnetic field of 300 mT for the unstructured and structured samples. The optical transmittance under magnetic field is normalized with respect to that obtained under non-magnetic field (*T* = *I/I*_0_, *T* is the normalized transmittance under magnetic field, *I* is the intensity of the transmitted light under magnetic field, *I*_0_ is the intensity of the transmission light under non-magnetic field). For the Fe_3_O_4_-PDMS composite films, applying the external magnetic field leads to the increased optical transmission. The structured films exhibit larger increases in the optical transmittance than the unstructured films with the same Fe_3_O_4_ content. The maximum enhancement in optical transmittance was seen from the structured film containing 1 wt% of Fe_3_O_4_ nanoparticles. However, it was found that the application of an external magnetic field reduces the optical transmission for the Fe-PDMS composite films with 3, 5, 7, and 10 wt% Fe nanoparticles. The unstructured films demonstrate larger change in the optical transmittance than the structured films with the same Fe content, which is different from the Fe_3_O_4_-PDMS composite films. The maximum change was observed from the unstructured film containing 7 wt% Fe nanoparticles.

When the external magnetic field is imposed on the composite films, the magnetic particles in the films will polarize and start to interact. Consequently, the particles will move in the PDMS matrix, trying to align with the external magnetic field to realize an energetically favorable configuration [30,31]. However, the particles can only move to some extent as they are connected with the elastic polymer matrix. The mechanical interactions between the particles and the matrix will cause the deformation of the composite films, namely, film magnetostriction.

The PDMS matrix is transparent, whereas the magnetic particles are opaque. Hence, the transmittance of the composite films depends on the area occupied by the PDMS matrix (geometric shadowing effect). We speculate that with an external magnetic field applied, the particle movement in the matrix could bring about a change in the particles’ overlap and a consequent variation in the film optical transmission because of a geometric shadowing effect of the optical path [32]. On the other hand, the film magnetostriction could change the film thickness and thus the film optical transmission.

**Figure 3:**
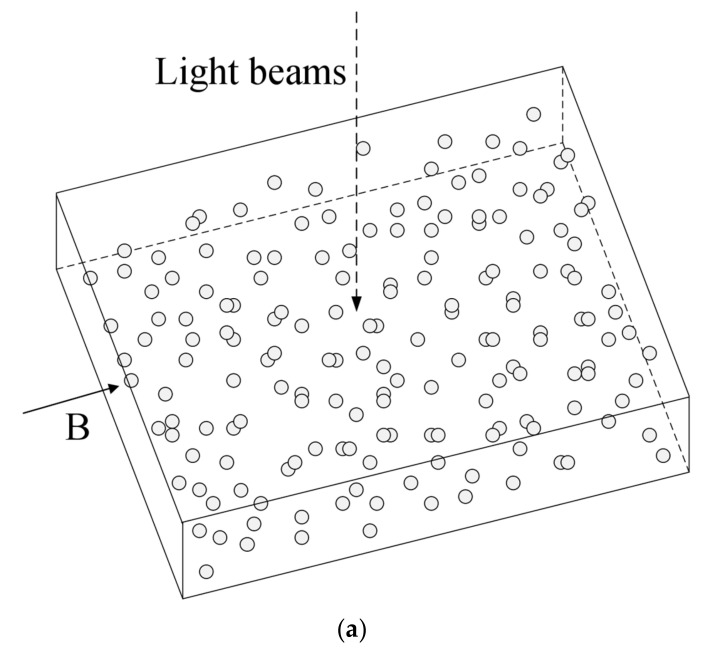

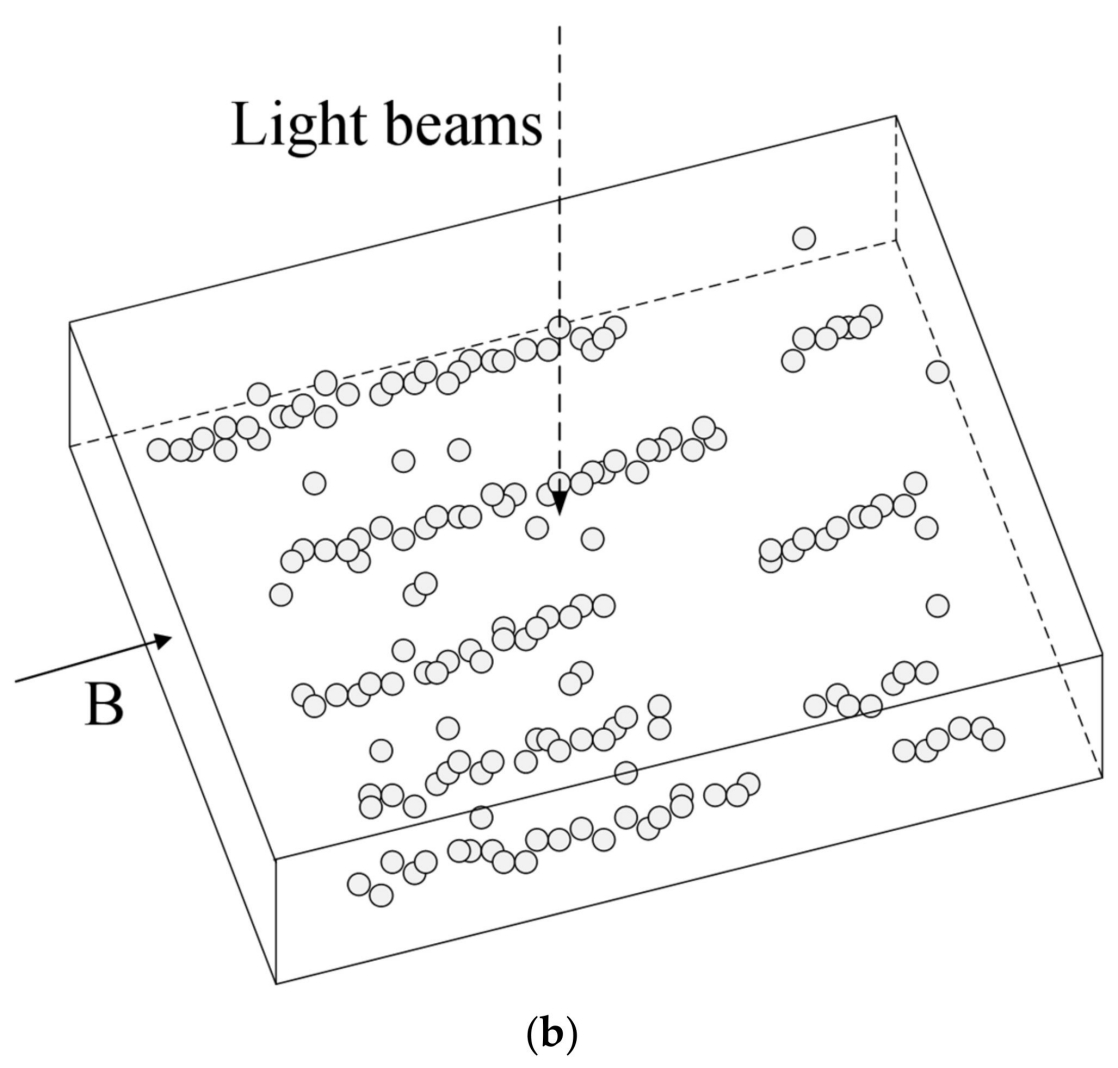
The representative volume elements for (**a**) unstructured and (**b**) structured films. **B** denotes the applied magnetic field.

A 3D numerical modeling process is carried out to investigate the effect of the particle movement in the PDMS matrix on the optical transmittance of the composite films when applying an external magnetic field. The representative volume element for the calculation is a cuboid PDMS matrix filled with 210 identical magnetic particles (Figure 3). For the realistic composite films, the magnetic nanoparticles are distributed in the PDMS matrix in the form of the agglomerates. Therefore, the magnetic particle diameter is chosen to be 2 µm in the modeling, which is comparable to the size of the agglomerated nanoparticles in the realistic samples. Each particle is glued to the PDMS matrix with no slipping. The PDMS matrix is considered as linear elastic isotropic material and its relative permeability is set to 1. The magnetic particles are assumed to be much more rigid than the PDMS matrix (i.e., the elastic modulus of the magnetic particles is much larger than that of the PDMS matrix). Hence, the influence of the elastic modulus of the magnetic particles on the calculation results is negligible. The composite film is considered as an incompressible material. The boundary conditions for the modeling are imposed in such a way that the bottom surface of the cuboid is fixed and the five other surfaces are free, which are identical with the experimental situation for the measurement of the film optical transmittance. In addition, the mesh is rendered sufficiently dense so that there are enough nodes inside each magnetic particle.

**Figure 4:**
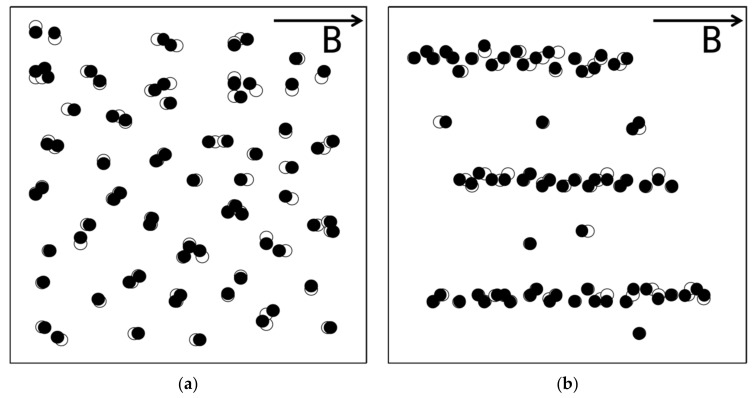
The two dimensional projections of the representative volume elements for (**a**) unstructured and (**b**) structured films on a surface perpendicular to the light beams used in the transmittance testing under an external magnetic field.

Both the unstructured and structured composite films were used in the numerical modeling. A homogenous magnetic field was applied to the films in the direction shown in Figure 3. The orientation of the light beams is also shown in the figure, which went through the composite films during the optical transmittance testing. The calculation results are displayed via the two dimensional projection of the system on a plane perpendicular to the light beams (Figure 4), where the open circles represent the initial positions of the magnetic particles, i.e., the positions before their movement under the action of the magnetic field. As is seen from Figure 4, for both types of films the particle movement leads to the separation of some overlapping particles and the overlapping of some separate particles. On the whole, however, the particle movement under magnetic field only causes a slight change in the film microstructure, namely, a slight change in the area occupied by the PDMS matrix. As a consequence, the film optical transmission changes slightly from the geometric shadowing effect.

In order to investigate the influence of the film magnetostriction on the film optical transmittance, a 2D numerical analysis is performed on a rectangular PDMS matrix filled with 100 identical magnetic particles. Here, the rectangle represents the cross-section of the film, in which the top side is the film surface, the bottom side is connected with the substrate, and the distance between the top side and the bottom side is the film thickness. The settings, definitions and parameters for the materials are the same as the above 3D simulation. As for the boundary conditions, the bottom side of the rectangular model is fixed and the other sides are free to move, which are identical with the experimental situation for measuring the film optical transmission.

**Figure 5:**
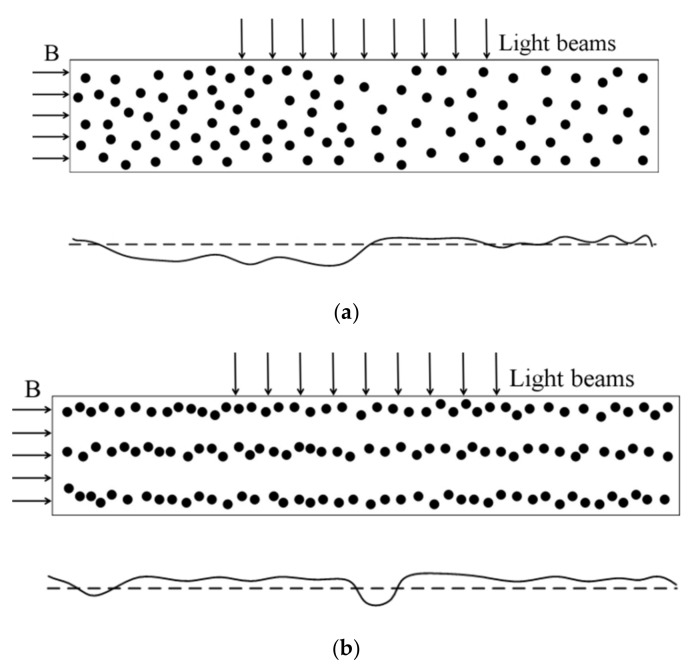
2D finite element model (up) and the calculated deformation of the film surface (down): (**a**) unstructured film, and (**b**) structured film.

Both samples, i.e., the unstructured and structured composite films, were used to perform the calculation. A homogenous magnetic field was imposed on the films in the orientation parallel to the fixed side, while the light beams for the optical transmittance testing went through the films in the direction perpendicular to the fixed side. The simulation results are presented in Figure 5, where the dashed line and the solid line represent the original surface and the deformed surface of the films, respectively. As is seen, the application of an external magnetic field brings about the film deformation and a consequent variation in the film thickness. The intensity of transmitted light decreases exponentially with the increasing film thickness, which can be expressed by

(2) $$I\;\propto \;\exp\left(-L\right),$$

where I and L denote the intensity of transmitted light and the film thickness, respectively [32,33]. A slight change in the film thickness will give rise to a remarkable variation in the film optical transmission. We therefore believe that the magnetically induced optical transmission of the composite films can be mainly ascribed to the variation in the film thickness resulting from the film magnetostriction. This hypothesis was verified by measuring the film thickness of the sample without and with a magnetic field, which was performed using an ellipsometer. It was found that applying an external magnetic field changes the thickness of the composite films. As an example, a decrease of ~350 nm in the film thickness was observed from the unstructured Fe_3_O_4_-PDMS sample containing 1 wt% Fe_3_O_4_ nanoparticles.

The simulation results also indicate both how the film thickness changes (contraction or stretch) and how much the film thickness changes depending on the specific way in which the magnetic particles are arranged in the PDMS matrix (film microstructure). This observation coincides with knowledge stated in the literature, i.e., MRE magnetostriction is greatly affected by its microstructure [25,27,34]. In this study, the film microstructure is randomly formed via spin casting. This might be an important reason why there is not any clear trend that is observed for the concentration in Figure 2, although the concentration has a pronounced effect on MRE magnetostriction [35,36].

**Figure 6:**
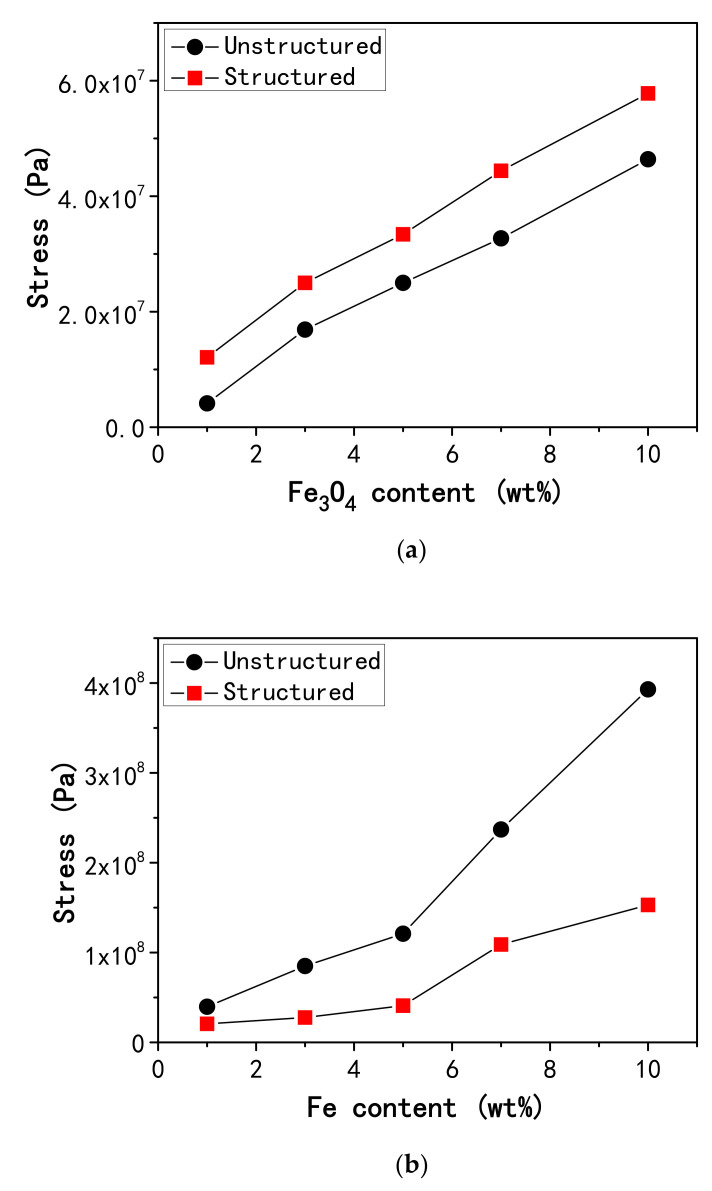
The film magnetostrictive stresses for the unstructured and structured samples with varied weight fractions of magnetic nanoparticles: (**a**) Fe_3_O_4_-PDMS films, and (**b**) Fe-PDMS films.

To further investigate the origin of the magnetically induced optical transmission of the composite films comprised of PDMS and magnetic nanoparticles, we measured the film magnetostrictive stresses for all samples under study that include the unstructured and structured films with varied Fe_3_O_4_ or Fe nanoparticle weight fractions of 1, 3, 5, 7, and 10 wt%. Figure 6 displays the measurements of the film magnetostrictive stresses. As shown in the figure, the film magnetostrictive stress increases with the concentration of the magnetic nanoparticles for the samples with the same film microstructure (unstructured or structured nanoparticle distributions) and nanofiller particles (Fe_3_O_4_ or Fe nanoparticles). It also can be seen from Figure 6 that the magnetostrictive stress in the structured film is greater than that in the unstructured film with the same nanoparticle content for the Fe_3_O_4_-PDMS samples. However, the film magnetostrictive stress in the unstructured film is observed to exceed that in the structured film with the same nanoparticle loading in the case of the Fe-PDMS samples.

Applying an external magnetic field will bring about a change in the shapes and dimensions of MRE samples, which is referred to as the magnetostrictive effect. In the self-consistent local field approximation, the magnetostrictive stresses parallel (*x*-axis) and perpendicular (*y*-axis) to the applied magnetic field are described by

(3) $$\sigma_{x}=-\frac{1}{2}{µ}_{eff}{µ}_{0}H_{0}^{2}(1+\gamma_{xx}),\;\sigma_{y}=+\frac{1}{2}{µ}_{eff}{µ}_{0}H_{0}^{2}(1-\gamma_{xy}).$$

Herein, *µ_eff_* is the effective relative composite permeability, *µ*_0_ is the permeability of free space, *H*_0_ is the intensity of the applied magnetic field, and both *γ_xx_* and *γ_xy_* are the magnetostriction coefficients [34]. *µ_eff_* depends on the relative particle permeability, the composite structure, and the particle volume fraction. The magnetostriction coefficients are defined by the strain derivatives. It is shown in Equation (3) that there are both contraction and stretch in the MRE sample when it is subjected to an external magnetic field. The theory predicts a contraction along the applied field and a stretch perpendicular to the field. For this work, the sample contraction might lead to a decreased film thickness, while the sample stretch probably brings about an increased film thickness.

The experimental results reveal that the application of an external magnetic field will produce stresses in the composite films consisting of PDMS and magnetic nanoparticles, which verifies that there is magnetostriction in the composite films since stress is closely related to strain (magnetostriction), as shown in the above magnetostriction theory. Therefore, the experimental results confirm the simulation result obtained in this study, i.e., the film magnetostriction results in the magnetically induced optical transmission of the composite films. Besides, according to the above magnetostriction theory, the film magnetostrictive stresses depend on the composite structure that includes the distance between a pair of particles in the composite, the angle of their line of centers to the direction of the applied field, and so on, which is different between Fe_3_O_4_-PDMS films and Fe-PDMS films. This might be an important reason for the disparity between them that is demonstrated in Figure 6, i.e., the magnetostrictive stress in the structured Fe_3_O_4_-PDMS film is larger than that in the unstructured counterpart with the same Fe_3_O_4_ content, while the magnetostrictive stress in the unstructured Fe-PDMS film is greater than that in the structured counterpart with the same Fe concentration.

In addition, we compared the measurements of the film magnetostrictive stresses with the variations in film optical transmittance resulting from the magnetic field. For the Fe_3_O_4_-PDMS films, the structured sample exhibits both a greater change in optical transmittance and a larger film stress than the unstructured counterpart with the same Fe_3_O_4_ content when the films are subjected to the external magnetic field. As an example, the normalized transmittance under magnetic field and the film magnetostrictive stress for the structured sample containing 1 wt% Fe_3_O_4_ are 1.30 and 1.21 × 10^7^ Pa, respectively, while the transmittance and stress values for the unstructured counterpart with the same Fe_3_O_4_ content are 1.23 and 4.13 × 10^6^ Pa, respectively. In the case of the Fe-PDMS films, both the greater change in optical transmittance and the larger film stress are observed from the unstructured sample rather than the structured counterpart with the same nanoparticle loading when the external magnetic field is applied.

The MRE microstructure has a significant effect on the sample magnetostriction, which results from the manufacturing process. In this work, there are two types of film microstructures. The structured sample has a microstructure with the particle columnlike structures in the PDMS matrix, while the unstructured film has a microstructure with the particle fractal structures randomly dispersed in the PDMS matrix. The above comparison suggests that there might be some relationship between the magnetically induced optical transmission and the film magnetostrictive stress under certain conditions since both of them are affected by the film microstructure according to the experimental results and the theoretical analysis.

## 4. Conclusions

The origin of the magnetically induced optical transmission of the composite films consisting of PDMS and magnetic nanoparticles is studied experimentally and theoretically. Applying an external magnetic field significantly varies the optical transmittance of the composite films, which is attributed to the film magnetostriction. This finding is of significance because it suggests that the composite films are potentially capable of being used for the magnetically modulated optical components. The future challenges are to investigate the variation of the film optical transmittance with the intensity of the applied magnetic field, as well as the correlation between the film magnetostriction and the particle arrangement in the PDMS matrix.
